# A novel computational approach to approximate fuzzy interpolation polynomials

**DOI:** 10.1186/s40064-016-3077-5

**Published:** 2016-08-27

**Authors:** Ahmad Jafarian, Raheleh Jafari, Maysaa Mohamed Al Qurashi, Dumitru Baleanu

**Affiliations:** 1 Department of Mathematics, Urmia Branch, Islamic Azad University, Urmia, Iran; 2 Departamento de Control Automático, CINVESTAV-IPN (National Polytechnic Institute), Mexico City, Mexico; 3 Department of Mathematics, King Saud University, Riyadh, 11495 Saudi Arabia; 4 Department of Mathematics, Faculty of Art and Sciences, Cankaya University, 06530 Balgat, Ankara, Turkey; 5 Institute of Space Sciences, Magurele-Bucharest, Romania

**Keywords:** Fuzzy neural networks, Fuzzy interpolation polynomial, Cost function, Learning algorithm

## Abstract

This paper build a structure of fuzzy neural network, which is well sufficient to gain a fuzzy interpolation polynomial of the form $y_{p}=a_{n}x_{p}^n+ \cdots +a_{1}x_{p}+a_{0}$ where $a_{j}$ is crisp number (for $j=0,\ldots ,n)$, which interpolates the fuzzy data $(x_{j},y_{j})\,(for\,j=0,\ldots ,n)$. Thus, a gradient descent algorithm is constructed to train the neural network in such a way that the unknown coefficients of fuzzy polynomial are estimated by the neural network. The numeral experimentations portray that the present interpolation methodology is reliable and efficient.

## Background

Artificial neural networks (ANNs) are mathematical or computational models based on biological neural networks. Neural networks consist of universal approximation potentiality, and they function best when the system has a high endurance to error when used to model. Recently, there have been rapid growth of ANNs which was utilized in various fields (Abbasbandy and Otadi 2006; Chen and Zhang 2009; Guo and Qin 2009; Jafarian and Jafari 2012; Jafarian et al. 2015a, b; Jafarian and Measoomynia 2011, 2012; Song et al. 2013; Wai and Lin 2013). One of the vital roles of ANN is finding FIPs as it proposed in this research.

Interpolation theory is one of the basic tool in applied and numerical mathematics. Interpolation has been used extensively, because it is one of the noteworthy techniques of function approximation (Boffi and Gastaldi 2006; Mastylo 2010; Rajan and Chaudhuri 2001). Using Newton’s divided difference scheme, a new technique was established in Schroeder et al. (1991) for polynomial interpolation. The problem related to multivariate interpolation has grabbed the attention of researchers world wide (Neidinger 2009; Olver 2006). There are various multivariate interpolation methods. In Olver (2006) they used a multivariate Vandermode matrix and its LU factorization, and Neidinger (2009) utilized the Newton-form interpolation. We recall that sparse grid interpolation is a further technique. In recent years this procedure is widely executed for the provision of an average approximation to a smooth function (Xiu and Hesthaven 2005). Utilizing the Lagrange interpolating polynomials, this approach introduces a polynomial interpolant on the basis of amounts of the function at the points in an amalgamation of product grids of minute dimension (Barthelmann et al. 2000). Existing trends on interpolation networks, have been revealed in Llanas and Sainz (2006), Sontag (1992). Numerable proof based on the notation that single hidden layer FNNs taking into account $m+1$ neurons, is able to learn $m+1$ isolated data $(x_{i},f_{i})\,\,(for\,i=0,\ldots ,m)$ with zero error has been established in Ito (2001). The detailed introduction and survey of major results can be extracted from Refs. Szabados and Vertesi (1990), Tikhomirov (1990).

This paper is inclined to the motive in order to deliver a fuzzy modeling technique by the utilization of FNNs for finding a FIP of the form

1 $$y_{p}=a_{n}x_{p}^n+ \cdots +a_{1}x_{p}+a_{0},$$

where $a_{j}\,\epsilon\,{\mathbb {R}}\,\,(for\,j=0,\ldots ,n)$, which interpolates the fuzzy data $(x_{j},y_{j})\,(for\,j=0,\ldots ,n)$. The proposed network is a formation, abiding of three layers whereas the extension principle of Zadeh (2005) elaborately describes the input-output connection of each unit. In the latest model, the unrevealed coefficients of fuzzy polynomial can be approximated by employing a cost function. Moreover, a learning technique which is associated with gradient decent procedure is formulated for the adjustment of connection weights to any achievable degree of precision.

This paper starts with a summary explanation of fuzzy numbers and fuzzy interpolation, then we provide the method of FNN for finding the crisp solution of the FIP. Two numerical examples are proposed to establish the validity and performance of the justified approach in “Numerical examples” section. Finally, “Concluding remarks” section presents the conclusions.

## Method description

Basically, the interpolation theory has a wide range of applications in mathematical analysis. In numerical analysis, the interpolation is a method or operation of finding from a few given terms of a series, as of numbers or observations, other intermediate terms in conformity with the law of the series. Generally, the interpolation techniques are in phase with elementary model of an interpolating function which can be stated as:

2 $$\begin{aligned}&s: {\mathbb {R}}\rightarrow {\mathbb {R}},\nonumber \\&s(x)= \sum _{j=1}^{n} y_{j}\cdot \phi _{j}(x), \end{aligned}$$

with the basis function $\phi _{j}(x): {\mathbb {R}}\rightarrow {\mathbb {R}}$ that elates in interpolation criteria:

$$\begin{aligned} \phi _{j}(x_{k})= \left\{ \begin{array}{ll} 1, &{}\quad \hbox {for}\,k=j, \\ 0, &{}\quad \hbox {for}\,k\ne j. \\ \end{array}\right. \end{aligned}$$

Suppose that ${\hat{x}}_{1}, \ldots ,\hat{x}_{n}$ be *n* fuzzy points in $E^{n}$ whereas, a fuzzy number ${\hat{y}}_{j}\in E$ is in direct relation with each ${\hat{x}}_{j}$ for $j=1,\ldots ,n.$ The sought function can be portrayed as follows:

3 $${\hat{s}}: E^{n}\rightarrow E: {\hat{s}}\left( \hat{x}\right) = \sum _{j=1}^{n} {\hat{y}}_{j}\cdot {\hat{\phi }}_{j}\left( \hat{x}\right) ,$$

where the ${\hat{\phi}}_{j}: E^{n}\rightarrow E$ for $j=1,\ldots ,n$ exhibit fuzzy functions that compensate the stipulation of interpolation:

$$\begin{aligned} {\hat{\phi }}_{j}\left( {\hat{x}}_{k}\right) = \left\{ \begin{array}{ll} 1, &{}\quad \hbox {for}\, k=j, \\ 0, &{}\quad \hbox {for}\, k\ne j. \\ \end{array}\right. \end{aligned}$$

### Fuzzy interpolation polynomial

**Fig. 1:**
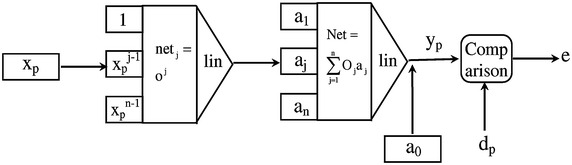
Fuzzy neural network equivalent to fuzzy interpolation polynomial

The interested are vested in finding FIP of the form

4 $$y_{p}=a_{n}x_{p}^n+ \cdots +a_{1}x_{p}+a_{0},$$

where $a_{j} \,\epsilon\,{\mathbb {R}}\,(for\,j=0,\ldots ,n)$, that interpolates the fuzzy data $(x_{j},y_{j})\,\,(for\,j=0,\ldots ,n)$. Taking into account a three layer FNN construction which is displayed by Fig. 1. The input-output connection of each unit of the offered neural network can be portrayed as mentioned below, when the $\alpha$-level sets of the fuzzy input $x_{p}$ is nonnegative, i.e., $0\le [x_{p}]_{l}^{\alpha }\le [x_{p}]_{u}^{\alpha }$:

- *Input unit*
  5 $$[o]^{\alpha }=\left[ [x_{p}]^{\alpha }_{l},[x_{p}]^{\alpha }_{u}\right] ,\quad p=0, \ldots ,n.$$
- *Hidden units*
  6 $$[O_{j}]^\alpha =f\left( \left[ [net_{j}]_l^\alpha ,[net_{j}]_u^\alpha \right] \right) = \left( \left( [o]^{\alpha }_{l}\right) ^{j},\left( [o]^{\alpha }_{u}\right) ^{j}\right) ,\quad j=1,\ldots ,n.$$
- *Output unit*
  7 $$\begin{aligned}{}[y_{p}]^{\alpha }&= {} F\left( [Net]_l^{\alpha }+a_{0},[Net]_u^{\alpha }+a_{0}\right) \nonumber \\&= {} \left( [Net]_l^{\alpha }+a_{0},[Net]_u^{\alpha }+a_{0}\right) ,\quad p=0,\ldots ,n, \end{aligned}$$
  We have
  $$[Net]_l^{\alpha }=\sum _{j\epsilon M}[O_{j}]_{l}^{\alpha } \cdot a_{j}+\sum _{j\epsilon C}[O_{j}]_{u}^{\alpha }\cdot a_{j},$$
  and
  $$[Net]_u^{\alpha }=\sum _{j\epsilon M}[O_{j}]_{u}^{\alpha } \cdot a_{j}+\sum _{j\epsilon C}[O_{j}]_{l}^{\alpha }\cdot a_{j},$$
  where $$M=\{a_{j}\ge 0\}, C=\{a_{j}< 0\}$$ and $$M\cup C=\{1,\ldots , n\}$$.

### Cost function

The input signals $x_p\ (\hbox {for}\,p = 0, \ldots , n)$ are represented to the network and then $y_n(x_p)$ which is an representing the network output upon the presentation of $a_j\ (\hbox {for}\,j = 0, \ldots , n)$, is calculated. Defining of cost function over the model parameters makes it a good forecaster. The mean squared error is termed to be as one of the vastly popular usable cost function. Now, let the $\alpha$-level sets of the fuzzy target output $d_{p}$ are exhibited as:

$$[d_{p}]^{\alpha }=\left[ [d_{p}]^{\alpha }_{l},\quad [d_{p}]^{\alpha }_{u}\right] ,\quad \alpha \in [0,1],$$

A cost function which is required to be diminished is stated for each $\alpha$-level sets as depicted:

8 $$e_{p}(\alpha )=e_{p}^{l}(\alpha )+e_{p}^{u}(\alpha ),\quad p=0,\ldots ,n,$$

where

9 $$e_{p}^{l}(\alpha )= \alpha \cdot \frac{\left( [d_{p}]^{\alpha }_{l}-[y_{p}]^{\alpha }_{l}\right) ^2}{2},$$

10 $$e_{p}^{u}(\alpha )= \alpha \cdot \frac{\left( [d_{p}]^{\alpha }_{u}-[y_{p}]^{\alpha }_{u}\right) ^2}{2}.$$

Generally the summed up error of the suggested neural network is extracted by:

11 $$e=\sum _{\alpha }\sum _{p=0}^n e_{p}(\alpha ).$$

Obviously, $$e\longrightarrow 0$$ means $$[y_{p}]^{\alpha }\longrightarrow [d_{p}]^{\alpha }$$.

### Fuzzy neural network learning approach

Suppose connection weights $a_{j}\,(for\,j=0,\ldots ,n)$ are randomly actuated by crisp numbers. Now tweaked rule is illustrated as (Ishibuchi et al. 1995):

12 $$\begin{aligned} a_{j}(t+1)&= {} a_{j}(t)+\varDelta a_{j}(t),\nonumber \\ \varDelta a_{j}(t) &= {} -\eta \cdot \frac{\partial e_{p}(\alpha )}{\partial a_{j}}+\gamma \cdot \varDelta a_{j}(t-1), \end{aligned}$$

where *t* denotes the number of moderation, $\eta$ signifies the rate of learning and $\gamma$ implies as the stationary momentum term. We calculate $\frac{\partial e_{p}(\alpha )}{\partial a_{j}}$ as follows:

13 $$\frac{\partial e_{p}(\alpha )}{\partial a_{j}}=\frac{\partial e_{p}^{l}(\alpha )}{\partial a_{j}}+\frac{\partial e_{p}^{u}(\alpha )}{\partial a_{j}}.$$

Hence complexities lies in the calculation of the derivatives $\frac{\partial e_{p}^{l}(\alpha )}{\partial a_{j}}$ and $\frac{\partial e_{p}^{u}(\alpha )}{\partial a_{j}}$. So we have:

$$\begin{aligned} \frac{\partial e_{p}^{l}(\alpha )}{\partial a_{j}}&= {} \frac{\partial e_{p}^{l}(\alpha )}{\partial [y_{p}]_l^{\alpha }}\cdot \frac{\partial [y_{p}]_l^{\alpha }}{\partial [Net]_l^{\alpha }} \cdot \frac{\partial [Net]_l^{\alpha }}{\partial a_{j}}\\&= {} -\alpha \cdot \left( [d_{p}]_{\alpha }^{l}-[y_{p}]_{\alpha }^{l}\right) . \frac{\partial [Net]_l^{\alpha }}{\partial a_{j}},\quad j=1,\ldots ,n, \end{aligned}$$

and

$$\frac{\partial e_{p}^{l}(\alpha )}{\partial a_{j}}=\frac{\partial e_{p}^{l}(\alpha )}{\partial [y_{p}]_l^{\alpha }}\cdot \frac{\partial [y_{p}]_l^{\alpha }}{\partial a_{j}}=-\alpha \cdot \left( [d_{p}]_{\alpha }^{l}-[y_{p}]_{\alpha }^{l}\right) ,\quad j=0,$$

where

$$\begin{aligned} \frac{\partial [Net]_l^{\alpha }}{\partial a_{j}}=\left\{ \begin{array}{ll}{[O_{j}]_l^{\alpha }},&{}\quad a_{j}\ge 0,\\ \\ {[O_{j}]_u^{\alpha }},&{}\quad a_{j}<0, \end{array} \right. \end{aligned}$$

also we have

$$\begin{aligned} \frac{\partial e_{p}^{u}(\alpha )}{\partial a_{j}}&= {} \frac{\partial e_{p}^{u}(\alpha )}{\partial [y_{p}]_u^{\alpha }}\cdot \frac{\partial [y_{p}]_u^{\alpha }}{\partial [Net]_u^{\alpha }} \cdot \frac{\partial [Net]_u^{\alpha }}{\partial a_{j}}\\&= {} -\alpha \cdot \left( [d_{p}]_{\alpha }^{u}-[y_{p}]_{\alpha }^{u}\right) \cdot \frac{\partial [Net]_u^{\alpha }}{\partial a_{j}},\quad j=1,\ldots ,n, \end{aligned}$$

and

$$\frac{\partial e_{p}^{u}(\alpha )}{\partial a_{j}}=\frac{\partial e_{p}^{u}(\alpha )}{\partial [y_{p}]_u^{\alpha }}\cdot \frac{\partial [y_{p}]_u^{\alpha }}{\partial a_{j}}=-\alpha \cdot \left( [d_{p}]_{\alpha }^{u}-[y_{p}]_{\alpha }^{u}\right) ,\quad j=0,$$

where

$$\begin{aligned} \frac{\partial [Net]_u^{\alpha }}{\partial a_{j}}=\left\{ \begin{array}{ll}{[O_{j}]_u^{\alpha }},&{}\quad a_{j}\ge 0,\\ \\ {[O_{j}]_l^{\alpha }},,&{}\quad a_{j}<0, \end{array} \right. \end{aligned}$$

### Upper bound approximation

#### **Theorem 1**

*Suppose*$p: {\mathfrak{R}}\rightarrow {\mathfrak{R}}$*is a continuous function, hence for each compact set*$\vartheta \subset E_{0}$*(the set of all the bounded fuzzy set), and*$\psi >0,$*there are*$m\in N,$*and*$a_{0},a_{i}\in {\mathfrak{R}}, i=1,2,\ldots ,m,$*which imply*

14 $$\forall \hat{x}\in \vartheta \quad and\quad \forall \breve{x}\in {\mathfrak{R}},\quad d\left( p\left( \breve{x}\right) ,\sum _{i=1}^{m}p_{i}\left( \hat{x}\right) a_{i}+a_{0}\right) < \psi ,$$

*where*$\psi$*is a finite number.*

#### *Proof*

The proof of theorem can be followed from the below results. $\square$

If $p: {\mathfrak{R}}\rightarrow {\mathfrak{R}}$, by applying the methodology of the extension principle, *p* can be extended to the fuzzy function that denotes by $p: E_{0}\rightarrow E$ as follows:

15 $$\forall u\in E_{0},\quad p(u)(y)=\bigvee _{p\left( \hat{x}\right) =y} \left\{ u\left( \hat{x}\right) \right\} \quad y\in {\mathfrak{R}},$$

*p* is termed as expanded function. Also, $cc({\mathfrak{R}})$ implies the bounded set of closed intervals of ${\mathfrak{R}}$. clearly

16 $$u\in E_{0}\Longrightarrow \forall \alpha \in (0,1],\quad [u]^{\alpha }\in cc({\mathfrak{R}}).$$

Moreover

17 $$Supp(u)\in cc({\mathfrak{R}}).$$

Henceforth, we let

18 $$Supp(u)=[s_{1}(u),s_{2}(u)].$$

#### **Theorem 2**

*Suppose*$$p: {\mathfrak{R}}\rightarrow {\mathfrak{R}}$$*is a continuous function, hence for each compact set*$$\vartheta \subset E_{0}, \varrho >0$$*and arbitrary*$$\varepsilon >0,$$*exist*$$m\in N,$$*and*$$a_{0},a_{i}\in {\mathfrak{R}},$$$$i=1,2,\ldots ,m,$$*implicate*

19 $$\forall {\hat{x}}\in \vartheta ,\quad d\left( p\left( {\hat{x}}\right) ,\sum _{i=1}^{m}p_{i}\left({\hat{x}}\right) a_{i}+a_{0}\right) < \varrho ,$$

*where*$\varrho$*is a finite number. The bottom and top bounds of the*$\alpha$-*level set of fuzzy function diminish to*$\varrho,$*but the center goes to*$\varepsilon$.

#### *Proof*

Because $\vartheta \subset E_{0}$ is a compact set, and so by Lemma 3, it can be supposed that $V\subset {\mathfrak{R}}$ be the compact set associated to $\vartheta . \forall \varepsilon >0$, therefore by the final outcome in Cybenko (1989), exist $m\in N$, and $a_{0},a_{i}\in {\mathfrak{R}}, i=1,2,\ldots ,m$, which imply

20 $$\forall {\hat{x}}\in V,\quad \left| p(\hat{x})-\sum _{i=1}^{m}p_{i}(\hat{x})a_{i}+a_{0}\right| < \varepsilon ,$$

holds. Let $q(\hat{x})=\sum \nolimits _{i=1}^{m}p_{i}(\hat{x})a_{i}+a_{0}, \hat{x}\in {\mathfrak{R}}$, then

21 $$\forall \hat{x}\in V,\quad \left| p\left( \hat{x}\right) -q\left( \hat{x}\right) \right| < \varepsilon .$$

Theorem 4 implies the validity of (19). $\square$

#### **Lemma 3**

*If*$\vartheta \subset E_{0}$*be a compact set, hence*$\vartheta$*is uniformly support-bounded, i.e. exists a compact set*$V\subset {\mathfrak{R}},$*implicates*$\forall u\in \vartheta , \hbox {Supp}(u)\subset V$.

#### **Theorem 4**

*Supposing*$\vartheta \subset E_{0}$*be compact, V the corresponding compact set of*$\vartheta,$*and*$p,q: {\mathfrak{R}}\rightarrow \ {\mathfrak{R}}$*are the continuous functions that compensate the relation mentioned below*

22 $$\forall \hat{x}\in V,\quad \left| p\left( \hat{x}\right) -q\left( \hat{x}\right) \right| < k,\quad k>0.$$

*Then*$$\forall u\in \vartheta , \ d(p(u)-q(u))\le k.$$

#### *Proof*

See Liu (2000). $$\square$$

## Numerical examples

The following examples has been used to narrate the methodology proposed in this paper.

### *Example 5*

**Fig. 2:**
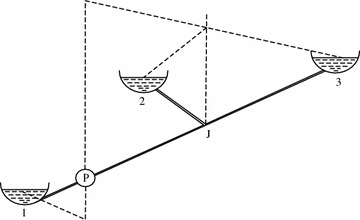
Pumping of water in order to transfer it from one tank to the further two tanks

The connection between three tanks and pipeline which is denoted by a constant *H* is represented by Fig. 2. It is a requirement to pump water in order to transfer it from one tank to the further two tanks. The mentioned system suffice the relation mentioned below

$$H=A_{0}\oplus A_{1}F_{1}\oplus A_{2}F_{2}\oplus A_{3}F_{3}$$

here $F_{1}=\sqrt{2x}, F_{2}=x\sqrt{x}, F_{3}=x^{3}$ are considered to be the flow quantity, where *x* is taken to be the elapsed time. The height of the pipe is mentioned by the term $H , A_{0}, A_{1}, A_{2}$ and $A_{3}$ are the pump characteristic coefficients, to be mentioned

$$A_{0}=2,\quad A_{1}=4,\quad A_{2}=3,\quad A_{3}=5$$

In below, four real uncertain data have been mentioned

$$x=\{ 6,(1,3,4),3,(2,3,4,6)\}$$

The iteration of data is continued for 19 times.

$$\begin{aligned}&H=\left\{ 1139.9472,(15.6568,162.3859,357.3137),\right. \\&\quad \quad \qquad \left. 162.3893, (58.4852,162.3859,357.3137,1139.9456)\right\} \end{aligned}$$

**Fig. 3:**
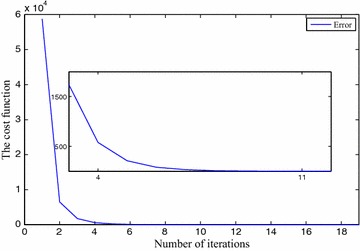
The error between the approximate solution and the exact solution

**Fig. 4:**
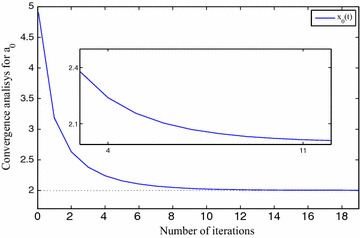
The approximated solution approaches to the exact one

**Fig. 5:**
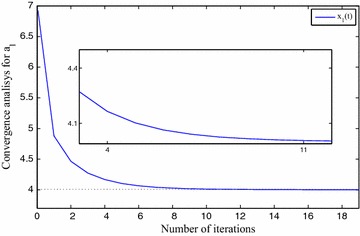
The approximated solution approaches to the exact one

**Fig. 6:**
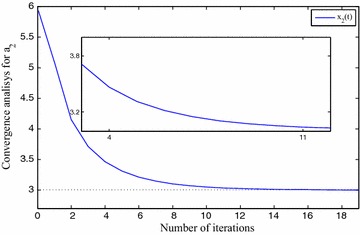
The approximated solution approaches to the exact one

**Fig. 7:**
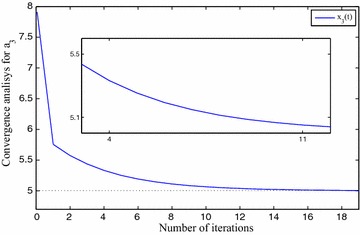
The approximated solution approaches to the exact one

We use $x_0=5, x_1=7, x_2=6, x_3=8, \eta =1\times 10^{-2}$ and $\gamma =1\times 10^{-2}$ for FNN. The approximation results are depicted in Table 1. The precision level of the solutions $x_0(t), x_1(t), x_2(t)$ and $x_3(t)$ are shown in Fig. 3, *t* implies the iterative numbers. It is eminent that by incrementing the iterations, the cost function diminishes to zero. The convergency criteria of the approximated solutions are portrayed using Figs. 4, 5, 6 and 7. For the purpose of attaining the exact solutions, the iterations in the figures have to be increased.

**Table 1:** Neural network approximation for the coefficients

*t*	$x_0(t)$	$x_1(t)$	$x_2(t)$	$x_3(t)$	Error for FNN
1	4.9018	6.9215	5.9307	7.9121	58,756.65
2	4.5321	6.6450	5.5480	7.6010	6479.790
3	4.0231	6.2056	5.1250	7.2212	1741.483
4	3.6850	5.8401	4.7851	6.7945	577.7597
5	3.2032	5.4001	4.3365	6.3330	210.8822
$\vdots$	$\vdots$	$\vdots$	$\vdots$	$\vdots$	$\vdots$
15	2.0008	4.0007	3.0008	5.0006	0.366883
16	2.0007	4.0005	3.0006	5.0005	0.151818
17	2.0005	4.0004	3.0005	5.0003	0.062895
18	2.0004	4.0003	3.0004	5.0002	0.026075
19	2.0003	4.0002	3.0003	5.0001	0.010815

### *Example 6*

Contemplate the sequential interpolation points:

$$\begin{aligned}&((1, 2, 3);\quad (-54, -29, -12)),\quad ((3, 4, 6); (-177, -87, -54)),\\&\quad \qquad ((2, 3, 5); (-128, -54, -29)) \end{aligned}$$

The exact solution for the given problem can be stated as:

$$y=-4x^2-5x-3.$$

This constrained is resolved by utilizing the technique of neural network suggested in this context, assuming $x_0=-0.5, x_1=-2.5, x_2=-1.5, \eta =3\times 10^{-2}$ and $\gamma =3\times 10^{-2}$.

**Fig. 8:**
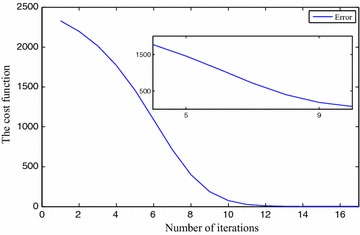
The error between the approximate solution and the exact solution

The approximation results are depicted in Table 2. The precision level of the solutions $x_0(t), x_1(t)$ and $x_2(t)$ are shown in Fig. 8, *t* implies the number of iterations.

**Table 2:** Neural network approximation for the coefficients

*t*	$x_0(t)$	$x_1(t)$	$x_2(t)$	Error for FNN
1	−0.5915	−2.5895	−1.5784	2330.5296
2	−0.9910	−2.9033	−1.9664	1896.6752
3	−1.3356	−3.3346	−2.3696	999.56201
4	−1.8050	−3.8798	−2.7561	401.56201
5	−2.2257	−4.1035	−3.1100	95.188500
$\vdots$	$\vdots$	$\vdots$	$\vdots$	$\vdots$
13	−2.9996	−4.9995	−3.9996	0.86688366
14	−2.9998	−4.9996	−3.9998	0.54635274
15	−2.9999	−4.9998	−3.9999	0.23614301
16	−3.0000	−4.9999	−4.0000	0.06896850
17	−3.0000	−5.0000	−4.0000	0.02003805

## Concluding remarks

This research introduces a new methodology in order to find a FIP which interpolates the fuzzy data $(x_j , y_j )\,\, (\hbox {for}\,j = 0, \ldots , n)$. To achieve this goal, a FNN equivalent to FIP was built, and a fast learning algorithm was defined for approximating the crisp unknown coefficients of the given polynomial. The proposed method was based on approximating FNN and the MATLAB software is used for the simulations. The innovative method was validated with two examples. The simulation results clearly illustrated the efficiency and computational advantages of the proposed technique. In particular, the error of approximation is minute.

## References

[CR1] Abbasbandy S, Otadi M (2006). Numerical solution of fuzzy polynomials by fuzzy neural network. Appl Math Comput.

[CR2] Barthelmann V, Novak E, Ritter K (2000). High dimensional polynomial interpolation on sparse grids. Adv Comput Math.

[CR3] Boffi D, Gastaldi L (2006). Interpolation estimates for edge finite elements and application to band gap computation. Appl Numer Math.

[CR4] Chen Lh, Zhang Xy (2009). Application of artificial neural networks to classify water quality of the yellow river. Fuzzy Inf Eng.

[CR5] Cybenko G (1989). Approximation by superpositions of a sigmoidal function. Math Control Signals Syst.

[CR6] Guo B, Qin L (2009). Tactile sensor signal processing with artificial neural networks. Fuzzy Inf Eng.

[CR7] Ishibuchi H, Kwon K, Tanaka H (1995). A learning of fuzzy neural networks with triangular fuzzy weghts. Fuzzy Sets Syst.

[CR8] Ito Y (2001). Independence of unscaled basis functions and finite mappings by neural networks. Math Sci.

[CR11] Jafarian A, Measoomynia S (2011). Solving fuzzy polynomials using neural nets with a new learning algorithm. Appl Math Sci.

[CR9] Jafarian A, Jafari R (2012) Approximate solutions of dual fuzzy polynomials by feed-back neural networks. J Soft Comput Appl. doi:10.5899/2012/jsca-00005

[CR12] Jafarian A, Measoomynia S (2012) Utilizing feed-back neural network approach for solving linear Fredholm integral equations system. Appl Math Model. doi:10.1016/j.apm

[CR10] Jafarian A, Jafari R, Khalili A, Baleanud D (2015). Solving fully fuzzy polynomials using feedback neural networks. Int J Comput Math.

[CR13] Jafarian A, Measoomy S, Abbasbandy S (2015). Artificial neural networks based modeling for solving Volterra integral equations system. Appl Soft Comput.

[CR14] Liu P (2000). Analyses of regular fuzzy neural networks for approximation capabilities. Fuzzy Sets Syst.

[CR15] Llanas B, Sainz FJ (2006). Constructive approximate interpolation by neural networks. J Comput Appl Math.

[CR16] Mastylo M (2010). Interpolation estimates for entropy numbers with applications to non-convex bodies. J Approx Theory.

[CR17] Neidinger RD (2009) Multivariable interpolating polynomials in newton forms. In: Joint mathematics meetings, Washington, DC, pp 5–8

[CR18] Olver PJ (2006). On multivariate interpolation. Stud Appl Math.

[CR19] Rajan D, Chaudhuri S (2001). Generalized interpolation and its application in super-resolution imaging. Image Vis Comput.

[CR20] Schroeder H, Murthy VK, Krishnamurthy EV (1991). Systolic algorithm for polynomial interpolation and related problems. Parallel Comput.

[CR21] Song Q, Zhao Z, Yang J (2013). Passivity and passification for stochastic TakagiSugeno fuzzy systems with mixed time-varying delays. Neurocomputing.

[CR22] Sontag ED (1992). Feedforward nets for interpolation and classification. J Comput Syst Sci.

[CR23] Szabados J, Vertesi P (1990). Interpolation of functions.

[CR24] Tikhomirov VM, Gamkrelidze RV (1990). Approximation theory, analysis II. Encyclopaedia of mathematical sciences.

[CR25] Wai RJ, Lin YW (2013). Adaptive moving-target tracking control of a vision-based mobile robot via a dynamic petri recurrent fuzzy neural network. IEEE Trans Fuzzy Syst.

[CR26] Xiu D, Hesthaven JS (2005). High-order collocation methods for differential equations with random inputs. SIAM J Sci Comput.

[CR27] Zadeh LA (2005). Toward a generalized theory of uncertainty (GTU) an outline. Inf Sci.

